# Adenosine A_2A_ Receptor Blockade Provides More Effective Benefits at the Onset Rather than after Overt Neurodegeneration in a Rat Model of Parkinson’s Disease

**DOI:** 10.3390/ijms25094903

**Published:** 2024-04-30

**Authors:** Ana Carla L. Nunes, Marta Carmo, Andrea Behrenswerth, Paula M. Canas, Paula Agostinho, Rodrigo A. Cunha

**Affiliations:** 1 CNC-Center for Neuroscience and Cell Biology, University of Coimbra, 3004-504 Coimbra, Portugal; aclnunes@gmail.com (A.C.L.N.); martacarmo@yahoo.com.br (M.C.); a.behrenswerth@web.de (A.B.); canas.paula@gmail.com (P.M.C.); pmgagostinho@gmail.com (P.A.); 2 Faculty of Medicine, University of Coimbra, 3004-504 Coimbra, Portugal

**Keywords:** adenosine, A_2A_ receptor, Parkinson’s disease, synapse, striatum, substantia nigra

## Abstract

Adenosine A_2A_ receptor (A_2A_R) antagonists are the leading nondopaminergic therapy to manage Parkinson’s disease (PD) since they afford both motor benefits and neuroprotection. PD begins with a synaptic dysfunction and damage in the striatum evolving to an overt neuronal damage of dopaminergic neurons in the substantia nigra. We tested if A_2A_R antagonists are equally effective in controlling these two degenerative processes. We used a slow intracerebroventricular infusion of the toxin MPP^+^ in male rats for 15 days, which caused an initial loss of synaptic markers in the striatum within 10 days, followed by a neuronal loss in the substantia nigra within 30 days. Interestingly, the initial loss of striatal nerve terminals involved a loss of both dopaminergic and glutamatergic synaptic markers, while GABAergic markers were preserved. The daily administration of the A_2A_R antagonist SCH58261 (0.1 mg/kg, i.p.) in the first 10 days after MPP^+^ infusion markedly attenuated both the initial loss of striatal synaptic markers and the subsequent loss of nigra dopaminergic neurons. Strikingly, the administration of SCH58261 (0.1 mg/kg, i.p. for 10 days) starting 20 days after MPP^+^ infusion was less efficacious to attenuate the loss of nigra dopaminergic neurons. This prominent A_2A_R-mediated control of synaptotoxicity was directly confirmed by showing that the MPTP-induced dysfunction (MTT assay) and damage (lactate dehydrogenase release assay) of striatal synaptosomes were prevented by 50 nM SCH58261. This suggests that A_2A_R antagonists may be more effective to counteract the onset rather than the evolution of PD pathology.

## 1. Introduction

Parkinson’s disease (PD) is characterized by slowed movements, rigidity, and impaired posture and balance at its onset, due to the degeneration of dopamine (DA) neurons in the substantia nigra and the loss of DA in their terminal fields mainly in the dorsal striatum [1]. PD is an evolving neurodegenerative disease, starting with a prodrome, followed by several clinically defined stages of increased seriousness [1]. The prodrome of PD [2] has been proposed to involve the initial degeneration of DA nerve terminals, followed by a back-dying process leading to the degeneration of nigra DA neurons and the onset of motor symptoms (reviewed in [3,4,5]). In fact, the onset of PD motor symptoms occurs when circa 30% of DA neurons in the nigra are lost, which is associated with a more profound loss (>50%) of DA in the striatum [6,7]. The mechanisms underlying this initial degeneration of striatal DA nerve terminals are still unclear, but increasing evidence obtained in animal models supports a role for aberrant corticostriatal plasticity and glutamatergic dysfunction in this prodromic phase of PD [8,9,10,11,12]. Accordingly, several genetic factors associated with familial forms of PD trigger an early modification of corticostriatal plasticity that precedes the onset of motor symptoms [13,14,15,16,17,18,19]. This conceptual framework prompts researchers to consider that the control of this initial striatal synaptotoxicity might be an effective strategy to facilitate the early arrest of the onset of motor symptoms characteristic of PD [4,5].

The adenosine modulation system has emerged in recent years as a candidate target to alleviate the burden of PD, leading to the FDA approval of adenosine A_2A_ receptor (A_2A_R) antagonists as the first nondopaminergic therapy for PD [20]. A_2A_R are particularly abundant in striatal medium spiny neurons of the indirect pathway, where they antagonize DA D_2_ receptor signaling and bolster the activity of this inhibitory pathway over-functioning in PD; this mechanism provides the rationale to understand the amelioration of motor function by the pharmacological or genetic blockade of A_2A_R in animal models of PD and PD patients (reviewed in [21,22]). A_2A_R also affords neuroprotection in PD models (reviewed in [23,24]) as well as in different brain diseases (reviewed in [25]). This A_2A_R-related neuroprotection in different brain regions that are devoid of the particularly high density of A_2A_R that is present in striatal medium spiny neurons depends on a different population of A_2A_R located in excitatory synapses, as concluded in animal models of Alzheimer’s disease [26,27] or convulsions [28] and possibly also in PD [29]. Accordingly, the neuroprotection and motor control afforded by A_2A_R antagonists in PD occur through different mechanisms [30], and synaptic A_2A_R display a gain of function in the PD prodrome controlling aberrant corticostriatal plasticity and the onset of motor symptoms in a 6-hydroxydopamine model of PD [12]. Additionally, studies in cultured neurons and cellular models exposed to toxins triggering PD-like symptoms showed that A_2A_R can also control the overt neurodegeneration of these cells [31,32,33]. However, it has not yet been explored if the neuroprotection afforded by A_2A_R antagonists in the context of PD is restricted to the prodrome and early PD when striatal synaptotoxicity is predominant, or if A_2A_R antagonists instead afford delayed neuroprotection, attenuating the dying-back mechanism that results in the delayed degeneration of the DA cell bodies in the substantia nigra.

To tackle this question, we resorted to an animal model based on the continuous infusion of MPP^+^ directly in the lateral ventricles lining the striatum [34] to trigger a slowly evolving PD-like neurodegeneration, where there is a temporal separation between the initial striatal synaptotoxicity and the delayed overt degeneration of DA cell bodies, with the aim of comparing the impact of A_2A_R antagonists on these two processes occurring in different phases of PD. 

## 2. Results

### 2.1. MPP^+^-Icv Infusion Triggers an Initial Loss of Striatal Dopamine at 25 Days Evolving to Nigra Dopamine Neuronal Damage Accompanied by Motor Deficits at 45 Days

**Figure 1:**
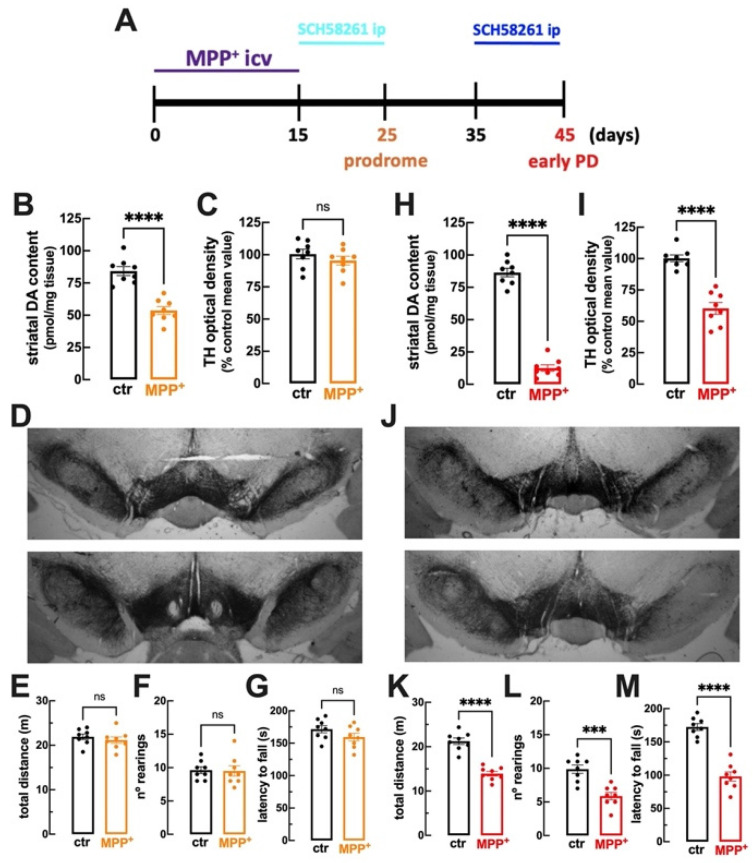
Rats challenged with an intracerebroventricular (icv) infusion of MPP^+^ (0.15 mg/kg/day) for 15 days displayed initial striatal toxicity without modifications of nigra neurodegeneration or motor deficits (after 10 days) followed by delayed nigra neurodegeneration and motor deficits (after 30 days): (**A**) Time course of experimental manipulations and analyses. At day 25 (10 days after stopping MPP^+^-icv infusion), compared to vehicle-infused rats (control—ctr, black bars), rats infused with MPP^+^ (orange) displayed decreased dopamine (DA) levels in the striatum (**B**) without modification either of tyrosine hydroxylase (TH) immunoreactivity in the nigra (**C**), as illustrated by the similar staining in vehicle-infused (**D**, top, 10× objective) and MPP^+^-infused (**D**, bottom, 10× objective), or of the total distance traveled in the open field (**E**), or of the number of rearing in the open field (**F**) and the latency to fall in the rotarod test (**G**). At day 45 (30 days after stopping MPP^+^-icv infusion), compared to vehicle-infused rats (control—ctr, black bars), rats infused with MPP^+^ (red) displayed a larger decrease in DA levels in the striatum (**H**) together with a reduction in TH immunoreactivity in the nigra (**I**), as illustrated by the similar staining in vehicle-infused (**J**, top, 10× objective), but decreased ipsilateral staining in MPP^+^-infused (**J**, bottom, 10× objective), as well as a decreased total distance traveled in the open field (**K**), a decreased number of rearing in the open field (**L**), and a shorter latency to fall in the rotarod test (**M**). Data are mean ± SEM; n = 8 mice per group; *** *p* < 0.001, **** *p* < 0.0001, Student’s *t*-test with Welsh correction; ns: nonsignificant (*p* > 0.05).

The MPP^+^-icv rat model of PD is characterized by unilateral striatal and nigra DA lesions [34,35], which are the neurochemical basis of motor deficits characteristic of PD [1]. We now report that the slow icv infusion of MPP^+^ for 15 days triggered an initial loss of striatal DA in the absence of motor alterations, typifying a prodrome phase of PD. Thus, compared to vehicle-icv-infused rats, we observed that 25 days after starting MPP^+^ infusion, rats displayed a reduction in the levels of DA in the ipsilateral dorsal striatum (Figure 1B; Table 1) but not in the contralateral dorsal striatum (n = 6), without a significant reduction in the density of TH (a marker of DA neurons) in the substantia nigra (Figure 1C,D; Table 1). This was accompanied by an unaltered motor function, as concluded from the lack of alterations of locomotion in the open field test (Figure 1E; Table 1), the lack of alterations in the number of rearing events in the open field (Figure 1F; Table 1), and a similar motor coordination performance in the rotarod test (Figure 1G; Table 1).

Later after MPP^+^-icv infusion, there was a larger extent of loss of striatal DA levels coupled to the overt neurodegeneration of DA neurons in the substantia nigra and motor dysfunction, typifying the onset of PD. Thus, compared to vehicle-icv-infused rats, we observed that 45 days after starting MPP^+^ infusion, rats displayed a substantial reduction in the levels of DA in the dorsal striatum (Figure 1H; Table 1) and a significant reduction in the density of TH in the substantia nigra (Figure 1I,J; Table 1). This was accompanied by the deterioration of motor function, as concluded from reduced locomotion in the open field test (Figure 1K; Table 1), a reduced number of rearing events in the open field (Figure 1L; Table 1), and reduced motor coordination in the rotarod test (Figure 1M; Table 1).

Thus, the slow icv infusion of MPP^+^ triggered a prodrome phase characterized by a selective loss of DA levels in the striatum without observable alterations in motor performance, followed by the later onset of PD characterized by motor dysfunction together with the overt neurotoxicity of DA neurons in the substantia nigra.

### 2.2. The Early Striatal Synaptotoxicity following MPP^+^-Icv Infusion Involves Dopaminergic and Glutamatergic Synapses

**Figure 2:**
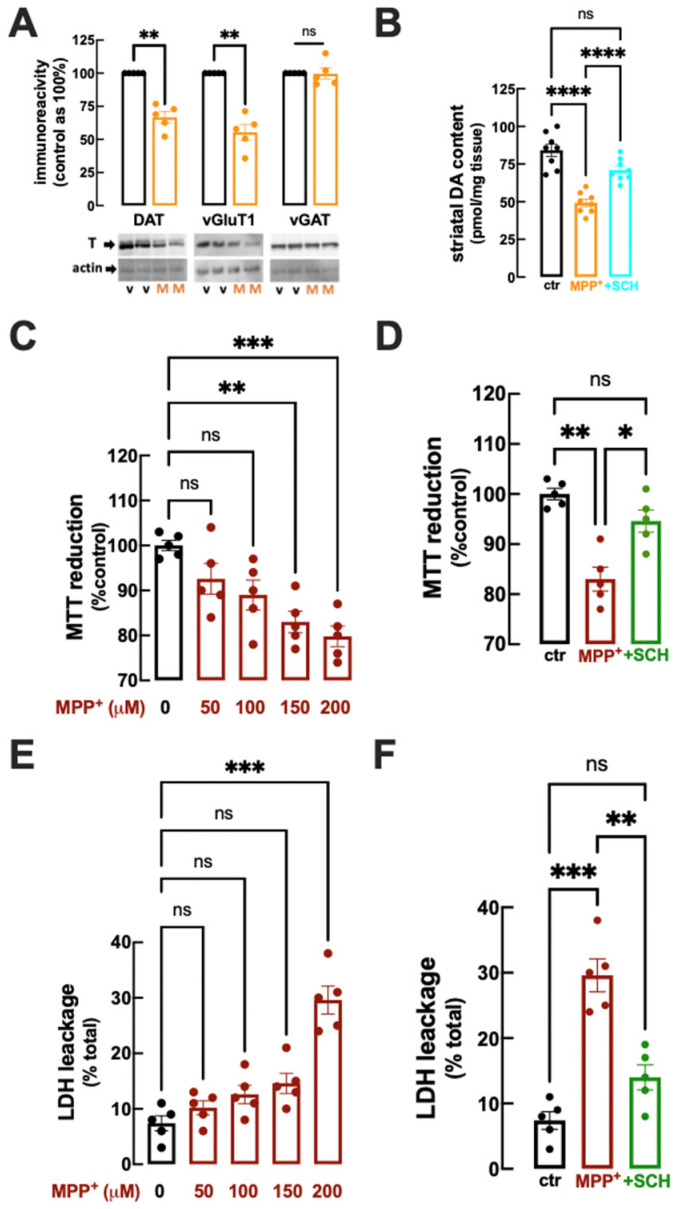
Rats challenged with an intracerebroventricular (icv) infusion of MPP^+^ (0.15 mg/kg/day) for 15 days displayed a loss of markers of dopaminergic and glutamatergic, but not GABAergic synapses, and this MPP^+^-induced striatal synaptotoxicity was attenuated by the A_2A_R antagonist SCH58261: (**A**) At day 25 (10 days after stopping MPP^+^-icv infusion), compared to vehicle-infused rats (black bars), rats infused with MPP^+^ (orange) displayed a decreased density of dopamine transporters (DATs) and vesicular glutamate transporter type 1 (vGluT1) but not vesicular GABA transporters (vGATs) in striatal membranes, as illustrated in the bar graph with average values and in the representative Western blot of DATs (top raw left, MW of 62 kDa), vGluT1 (top raw center, MW of 68 kDa) and vGATs (top raw right, MW of 57 kDa), normalized by re-probing with β-actin (lower raws, MW of 42 kDa), comparing controls (v-vehicle) with MPP^+^-challenged rats (M). (**B**) The daily administration of SCH58261 (0.1 mg/kg/day, i.p.) from day 15 to 24 (light blue bar and symbols; see time course in Figure 1A) attenuated the MPP^+^-induced decrease in dopamine (DA) levels in the striatum observed at day 25 (red bar and symbols). (**C**–**F**) Compared to control (ctr, no drugs), MPP^+^ (dark red bars and symbols) caused a concentration-dependent decrease in functionality, assessed as a reduction in MTT (**C**), and of the viability, assessed as the release of lactate dehydrogenase (LDH, **E**) and striatal synaptosomes, which were both prevented by 50 nM SCH58261 (**D**,**F**; green bar and symbols). Data are mean ± SEM; n = 5 mice per group in (**A**,**C**–**F**) and n = 8 mice per group in (**B**); * *p* < 0.05, ** *p* < 0.01, *** *p* < 0.001, **** *p* < 0.0001, Student’s *t*-test with Welsh correction in (**A**) and one-way ANOVA followed by Tukey post hoc tests in (**B**–**F**); ns: nonsignificant (*p* > 0.05).

In order to confirm that the loss of striatal DA levels corresponds to the loss of nerve terminals in the striatum, we carried out a comparative analysis of the density of markers of different nerve terminals in ipsilateral striatal membranes, namely DA synapses (DATs, DA transporters), corticostriatal synapses (vGluT1, vesicular glutamate transporter type 1) and GABAergic synapses (vGATs, vesicular GABA transporters), in a new group of vehicle- and MPP^+^-icv-infused rats. As shown in Figure 2A, compared to vehicle-icv-infused rats (control), we observed that rats sacrificed 25 days after starting MPP^+^ infusion displayed a significant reduction in the density of striatal DAT (66.7 ± 4.33% of control DAT immunoreactivity in MPP^+^-infused rats, n = 5, *p* = 0.002 with Student’s *t*-test) as well as a significant decrease in the density of striatal vGluT1 (55.4 ± 5.92% of control vGluT1 immunoreactivity in MPP^+^-infused rats, n = 5, *p* = 0.002 with Student’s *t*-test), but no significant alteration in striatal vGATs (99.7 ± 4.26% of control vGAT immunoreactivity in MPP^+^-infused rats, n = 5, *p* = 0.944 with Student’s *t*-test). Thus, the slow infusion of MPP^+^ triggers a prodrome phase characterized by a selective striatal synaptotoxicity involving both DA and glutamate terminals but not GABAergic terminals.

### 2.3. A_2A_R Blockade Alleviates Early Striatal Synaptotoxicity following MPP^+^-Icv Infusion

A_2A_R are mostly synaptic receptors throughout the brain [25] and control synaptotoxicity in different brain disease conditions such as hippocampal glutamatergic synapses in animal models of Alzheimer’s disease [26,27,36] or epilepsy [28]. Although A_2A_R are far more abundant in striatal medium spiny neurons than elsewhere in the brain [29], A_2A_R are also located in DAergic [37,38,39] and glutamatergic synapses in the striatum [40,41], prompting the hypothesis that these synaptic A_2A_R may play a relevant role in the control of the early synaptotoxicity observed in the PD prodrome. 

For this purpose, we gathered a new cohort of rats divided into three groups, namely vehicle-icv-infused, MPP^+^-icv-infused, and MPP^+^-icv-infused rats that were treated with the A_2A_R antagonist SCH58261 (0.1 mg/kg daily) from day 15 to day 24, with all mice sacrificed at day 25. We first confirmed that 25 days after starting MPP^+^ infusion, rats displayed a reduction in the levels of DA in the dorsal striatum compared to vehicle-icv-infused rats (86.2 ± 4.51 pmol/mg tissue for vehicle and 49.3 ± 2.46 pmol/mg tissue for MPP^+^, n = 8, *p* < 0.0001 with one-way ANOVA; Figure 2B). Notably, SCH58261 after MPP^+^-icv infusion displayed significantly preserved levels of striatal DA (71.1 ± 2.60 pmol/mg tissue for vehicle, n = 8, *p* < 0.0001 vs. MPP^+^-infused rats with no SCH58261 treatment, one-way ANOVA; Figure 2B).

We next prepared synaptosomes from the contralateral dorsal striatum of the vehicle-injected rats, with the aim of confirming if A_2A_R antagonists control the direct synaptotoxicity of MPP^+^. We first defined the concentration-dependent impact of the direct exposure of striatal synaptosomes to MPP^+^. As shown in Figure 2C, a 150 μM concentration of MPP^+^ significantly decreased the functionality of striatal synaptosomes, as concluded from their decreased ability to reduce MTT (83.0 ± 2.39% of control, n = 5, *p* = 0.003, one-way ANOVA). Notably, this MPP^+^-induced decrease in MTT reduction was prevented by 50 nM SCH58261 (94.6 ± 2.21% of control, n = 5, *p* = 0.020 vs. 150 μM MPP^+^ in the absence of SCH58261, one-way ANOVA; Figure 2D). 

We next confirmed this ability of A_2A_R to control MPP^+^-induced synaptotoxicity by quantifying lactate dehydrogenase (LDH) release as a direct index of synaptosomal damage. As shown in Figure 2E, a 200 μM concentration of MPP^+^ triggered a significant disruption of striatal synaptosomes, as concluded from the release of LDH (7.40 ± 1.36% of total LDH in the absence and 29.6 ± 2.50% in the presence of MPP^+^, n = 5, *p* < 0.0008 with one-way ANOVA). Again, this MPP^+^-induced release of LDH was prevented by 50 nM SCH58261 (14.0 ± 1.92% of total LDH, n = 5, *p* = 0.003 vs. 200 μM MPP^+^ in the absence of SCH58261, one-way ANOVA; Figure 2F). 

Collectively, these data confirm that A_2A_R blockade directly controls the precocious striatal synaptotoxicity upon exposure to MPP^+^, mimicking the PD prodromic phase.

### 2.4. A_2A_R Blockade during the Prodrome Is More Efficient than during Early PD to Prevent Behavioral and Neurochemical Features of PD

**Figure 3:**
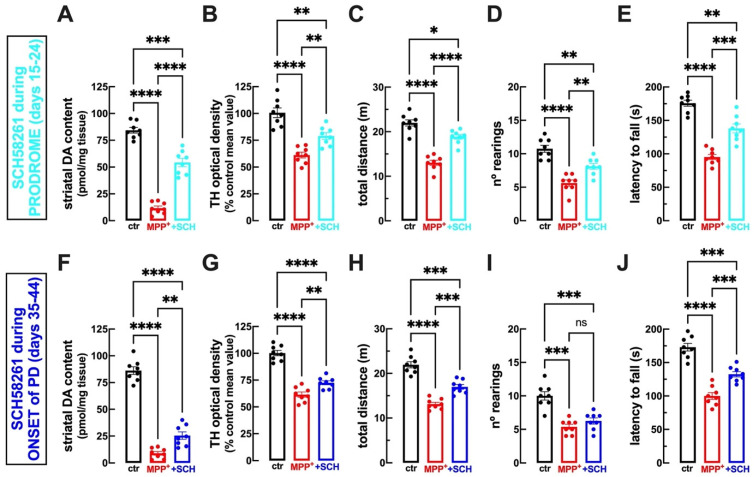
Blockade of A_2A_R during the prodrome of PD afforded more robust neuroprotection and alleviation of motor deficits than A_2A_R blockade during the onset of PD. As illustrated in the schematic time course of the experiments depicted in Figure 1A, rats were challenged with an intracerebroventricular (icv) infusion of vehicle (control-ctr, black bars, and symbols) or MPP^+^ (0.15 mg/kg/day) for 15 days and then treated with vehicle (red bars and symbols) or with the A_2A_R antagonist SCH58261 (0.1 mg/kg/day, i.p.) either during the prodromic phase (from day 15 to 24; top raw, (**A**–**E**), light blue bars and symbols) or during the onset of PD (from day 35–44; bottom raw, (**F**–**J**), dark blue bars and symbols). Rats were analyzed on day 45, first evaluating their behavior and, after sacrifice, their dopamine (DA) neurochemical profile in the striatum and tyrosine hydroxylase (TH) immunoreactivity in the substantia nigra: (**A**,**F**) striatal dopamine levels; (**B**,**G**) TH immunoreactivity in the substantia nigra; (**C**,**H**) total distance traveled in the open field; (**D**,**I**) number of rearing events in the open field test; (**E**,**J**) latency to fall in the rotarod test. Data are mean ± SEM of 8 mice per group; * *p* < 0.05, ** *p* < 0.01, *** *p* < 0.001, **** *p* < 0.0001, one-way ANOVA followed by Tukey post hoc tests; ns: nonsignificant (*p* > 0.05).

We next enquired if the ability of A_2A_R to control the initial MPP^+^-induced synaptotoxicity before the onset of motor symptoms translated into neuroprotection and the attenuation of motor symptoms that emerge later. Thus, rats were infused icv with MPP^+^, then treated with SCH58261 during the prodromic phase (between 15 and 24 days after starting MPP^+^ infusion), and were analyzed neurochemically and behaviorally at the onset of motor symptoms. At 45 days after starting MPP^+^-icv infusion, rats displayed a reduction in the levels of DA in the dorsal striatum compared to vehicle-icv-infused rats, which was significantly attenuated by SCH58261 treatment between days 15 and 24 (Figure 3A; Table 1). Rats exposed to MPP^+^ also displayed a significant reduction in the TH density in the substantia nigra, which was significantly attenuated by SCH58261 treatment between days 15 and 24 (Figure 3B; Table 1). Locomotion in the open field test was reduced in MPP^+^-treated rats, which was significantly attenuated by SCH58261 treatment between days 15 and 24 (Figure 3C; Table 1). MPP^+^-infused rats had a reduced number of rearing events in the open field, which was significantly attenuated by SCH58261 treatment between days 15 and 24 (Figure 3D; Table 1). Finally, MPP^+^-infused rats had reduced motor coordination in the rotarod test, which was significantly attenuated by SCH58261 treatment between days 15 and 24 (Figure 3E; Table 1).

We next tested if a delayed treatment with SCH58261 between 35 and 44 days after starting MPP^+^ infusion still afforded a behavioral and neurochemical benefit to the rats that were analyzed 45 days after starting MPP^+^ infusion. MPP^+^-treated rats displayed a reduction in the levels of DA in the dorsal striatum compared to vehicle-icv-infused rats, which was significantly attenuated by SCH58261 treatment between days 35 and 44 (Figure 3F; Table 1). However, the benefit afforded by this delayed SCH58261 treatment (21.3 ± 4.61% reduction, n = 8) was significantly lower (*p* < 0.0001) than the benefit afforded by the earlier SCH58261 treatment (62.4 ± 3.51% reduction, n = 8). MPP^+^ also triggered a significant reduction in the TH density in the substantia nigra, which was significantly attenuated by SCH58261 treatment between days 35 and 44 (Figure 3G; Table 1). However, the benefit afforded by this delayed SCH58261 treatment (28.5 ± 4.73% reduction, n = 8) was significantly lower (*p* = 0.026) than the benefit afforded by the earlier SCH58261 treatment (45.14 ± 4.71% reduction, n = 8). Locomotion in the open field test was reduced in MPP^+^-treated rats, which was significantly attenuated by SCH58261 treatment between days 35 and 44 (Figure 3H; Table 1). However, the benefit afforded by this delayed SCH58261 treatment (43.8 ± 6.82% reduction, n = 8) was significantly less (*p* = 0.020) than the benefit afforded by the earlier SCH treatment (65.4 ± 4.11% reduction, n = 8). MPP^+^-infused rats had a reduced number of rearing events in the open field, which was not significantly modified by SCH58261 treatment between days 35 and 44 (Figure 3I; Table 1), in contrast to the protection afforded by the earlier SCH58261 treatment. Finally, MPP^+^-infused rats had reduced motor coordination in the rotarod test, which was significantly attenuated by SCH58261 treatment between days 35 and 44 (Figure 3J; Table 1). However, the benefit afforded by this delayed SCH58261 treatment (44.5 ± 1.04% reduction, n = 8) was significantly less (*p* = 0.002) than the benefit afforded by the earlier SCH58261 treatment (53.2 ± 0.70% reduction, n = 8).

Thus, A_2A_R antagonists seem to have a dual impact on the evolving PD neuropathology and motor deficits, with a greater impact when applied earlier and attenuating synaptotoxicity, but also able to reduce the overt neurodegeneration of DA neurons when applied later.

## 3. Discussion

The present study shows that the slow icv infusion of MPP^+^ allows distinguishing an initial striatal synaptotoxicity without motor dysfunction that mimics a prodrome phase of PD from a delayed onset of motor symptoms coupled to the overt neurotoxicity of DA neurons in the substantia nigra that mimics the early phase of PD. The exploitation of this evolving PD model showed that the blockade of A_2A_R afforded a dual benefit, preventing both early striatal synaptotoxicity as well as delayed nigra neurotoxicity. However, the A_2A_R blockade afforded a more robust behavioral benefit when preventing early striatal neurotoxicity rather than delayed nigra neurotoxicity. 

The currently exploited model of evolving PD based on the slow icv infusion of MPP^+^ revealed an early striatal synaptotoxicity, typified by a reduction in the levels of striatal DA in the absence of any evident modification in the number of TH-immunopositive cell bodies in the substantia nigra. This is in accordance with the previously documented ability of MPP^+^ to directly affect nerve terminals (e.g., [42,43]), as confirmed by the decreased functionality and disruption of striatal synaptosomes exposed to MPP^+^. Importantly, the MPP^+^-induced striatal synaptotoxicity was observed before the onset of motor symptoms, which only emerged when a reduction in nigra DA cell bodies was observed. This time course of neurochemical/morphological and behavioral alterations supports the concept that striatal synaptotoxicity rather than the overt degeneration of nigra DA neurons may be a primary modification predating the motor clinical symptoms that define PD, as was proposed more than 10 years ago [44]. 

Indeed, alterations in striatal DA turnover [45], namely in twin studies [46], and a severe reduction in the staining of axon terminals in the putamen [47,48], predate the onset of clinical symptoms and the decreased immunostaining of DA neurons in the substantia nigra of PD patients. Furthermore, different genetic rodent PD models also support the contention that PD initially affects striatal dopaminergic projections before the occurrence of the overt degeneration of nigra DA cell bodies and the emergence of motor symptoms (reviewed in [49]). A similar pattern of initial damage in striatal dopaminergic terminals followed later by an overt loss of dopaminergic cell bodies in the nigra was also observed in toxicological models of slow onset of PD [12,50,51]. 

Importantly, our present analysis of markers of different nerve terminals revealed an initial decrease in both DAergic synaptic markers (DATs) as well as glutamatergic synaptic markers (vGluT1), but not GABAergic synaptic markers (vGATs). The reduction in DAT density is in line with the reduction in DA levels in the striatum at early time points after MPP^+^-icv administration, prompting the conclusion that MPP^+^ triggers an initial dysfunction and loss of DAergic synapses in the striatum. However, the observed decrease in vGluT1 density seems at odds with the observed early hyperexcitability of corticostriatal transmission in PD (reviewed in [52]). Nevertheless, previous studies have provided morphological evidence for a decreased density of corticostriatal glutamatergic synapses in PD [8,53,54] (but also see [55]) together with functional evidence of increased firing rates and bursting activity of the corticostriatal pathway [56,57,58,59,60]. Furthermore, DAergic deafferentiation leads to alterations in corticostriatal basal synaptic transmission and plasticity (e.g., [9,59,61,62]). These altered synaptic glutamate levels in the absence of DA control may contribute to abnormal corticostriatal plasticity [63] and the remodeling of spines in striatal medium spiny neurons associated with the emergence of PD motor symptoms [64]. This suggests that the initial striatal synaptotoxicity in the prodrome of PD might involve an intertwined alteration in decreased DAergic control and abnormal glutamatergic transmission in the striatum, contributing to the evolving pathophysiology of PD (reviewed in [63,64,65]). Thus, unraveling the mechanisms controlling this initial synaptotoxicity in the PD prodrome may be paramount to devise novel and effective strategies to prevent or delay the evolution of the purported dying-back neurodegeneration (reviewed in [49]) and the onset of PD motor symptoms.

We now report the ability of A_2A_R to control the initial striatal synaptotoxicity in the MPP^+^-icv model of early PD. Thus, the administration of a selective A_2A_R antagonist prevented the early striatal synaptotoxicity following MPP^+^-icv infusion, which afforded robust neuroprotection against the delayed neurodegeneration of DA neurons in the nigra and the associated motor dysfunction. This is in agreement with the previously reported ability of A_2A_R blockade to prevent the loss of DA in the striatum of mice displaying motor deficits upon challenge with 6-hydroxydopamine [66,67] or with MPTP [68,69]. We provide direct evidence that A_2A_R directly control striatal synaptotoxicity, by showing that MPP^+^-induced dysfunction and damage of striatal synaptosomes (purified synaptic fraction) is prevented by a selective A_2A_R antagonist. Although the mechanisms of the A_2A_R-mediated control of synaptotoxicity still need to be clarified [25], several studies in different models of brain diseases have reported the synaptoprotective effect of A_2A_R (e.g., [26,28,70,71,72]). 

In the striatum, A_2A_R are located in both glutamatergic [38,40,41,73] and DAergic terminals [37,38,39], and they control synaptic adaptive processes in the striatum upon DA depletion [74] or upon rotenone-induced toxicity [75]. In particular, it was previously reported that presynaptic A_2A_R are upregulated in a model of presymptomatic PD, and they control the aberrant corticostriatal plasticity that predates the onset of motor symptoms [12]. It is tempting to note that A_2A_R have been proposed to control striatal glutamatergic excitotoxicity [76], which may be a trigger of striatal synaptotoxicity in the prodrome of PD (reviewed in [63,64,65]). The interest in considering this synaptic A_2A_R-mediated control of excitotoxicity as a mechanism of PD evolution is bolstered by the recent claim that striatal DA deficits may not be sufficient to trigger the cascade of events leading to motor dysfunction [77], paving the way to incorporate the need for a simultaneous glutamatergic dysfunction, eventually resulting from the accumulation of α-synuclein [78,79], which is also controlled by A_2A_R [32,80]. This scenario does not exclude the possible additional or alternative involvement of an alteration in astrocytic function and/or microglia-derived neuroinflammation, two processes involved in the onset/evolution of PD (reviewed in [81,82]), both also controlled by A_2A_R [83,84].

Apart from this prominent role during the presymptomatic phase of PD that provided the more robust impact of A_2A_R on motor dysfunction, our present results also indicate that A_2A_R might additionally control the neurodegeneration of DA neurons in the nigra. In fact, the administration of the tested A_2A_R antagonist after the expression of synaptotoxic damage in the striatum still attenuated the decreased TH immunoreactivity in the substantia nigra. Although a direct effect of A_2A_R on the viability of DA neurons was not directly tested, previous studies have reported the ability of A_2A_R to control the damage of neurons [85,86,87,88,89], in particular mesencephalic neurons in culture [31], as well as the loss of viability of cells used as models of DA neurons such as differentiated human neuroblastoma SH-SY5Y cells exposed to MPP^+^ or 6-hydroxydopamine [33,90,91]. 

The present findings prompt us to conclude that the role of A_2A_R antagonists as anti-parkinsonian drugs [20] probably results from a combination of different mechanisms apart from the classical ability of A_2A_R to control D_2_ receptor function in medium spiny neurons of the indirect pathway [21,22,23,24]. In fact, our findings support the contention that targeting presynaptic A_2A_R controlling synaptotoxicity might be a main justification for the neuroprotection afforded by A_2A_R antagonists [29]. It still remains to be tested if this A_2A_R-mediated control of synaptotoxicity in the context of the PD prodrome is similar to that observed in different brain areas through mechanisms that are still unclear [25], or if it might involve the ability of A_2A_R to control α-synuclein [32,80] and its impact on the function and viability of striatal synapses [78,79] or an A_2A_R-mediated control of cholinergic interneurons [92,93,94]. Importantly, the benefits of A_2A_R antagonists might additionally involve the direct control of the loss of viability of nigra DA neurons [31] or control of astrocytes [84] or microglia-derived neuroinflammation [83]. All these mechanisms are complementary to the initially recognized A_2A_R-D_2_R interplay [21,22,23,24] and are probably engaged at different time periods during the evolution of PD and involve A_2A_R in different cellular and subcellular localizations. Given that A_2A_R in different localizations have a different interactome [95] and different pharmacological profiles [96], it becomes important to consider that different optimal doses of different A_2A_R antagonists may be required to target the different A_2A_R responsible for synaptoprotection, neuroprotection, and motor benefits [30], a concern critical for the adequate and effective exploitation of A_2A_R antagonists in PD. Finally, a future detailed exploration of the mechanisms of neurotoxicity in synapses and cell bodies may prompt additional strategies to bolster neuroprotection in the different phases of PD.

## 4. Materials and Methods

### 4.1. Animals

We used adult Wistar rats of both sexes (a total of 90 rats; 58 males and 32 females) with 3 months of age (221.4 ± 4.3 g) obtained from Charles River (Barcelona, Spain), which were housed in groups of 3–4 under controlled temperature (23 ± 1 °C) and a 12 h light/dark cycle, with free access to food and water. Rats were handled following European Community guidelines (EU Directive 2010/63/EU) and the Portuguese law on animal care (1005/92), and all procedures were approved by the Ethical Committee of the Center for Neuroscience and Cell Biology of Coimbra (ORBEA-128/2015). 

### 4.2. Intracerebroventricular Infusion of MPP^+^ to Model PD

Intracerebroventricular (icv) infusion was carried out as previously described [33,97]. After anesthesia with a combination of ketamine (100 mg/kg, i.p.) and xylazine (20 mg/kg, i.p.), MPP^+^ (5.5 mg/mL; Sigma, Sintra, Portugal) was administered (0.25 μL/h for 14 days, corresponding to a dose of 0.15 mg/kg/day) directly into the lateral ventricle through osmotic minipumps (Model 1002; Alzet, Cupertino, CA, USA), placed in a subcutaneous pocket in the dorsal region, and connected via polyethylene tubing to an intracranial cannula (Alzet Brain Infusion Kit II) targeting the following coordinates relative to bregma: 1.5 mm posterior, 1.0 mm lateral, and 3.7 mm below the horizontal plane of bregma. Control animals received a similar administration of saline, used as the vehicle.

### 4.3. Behavioral Analysis 

Behavioral tests were carried out from 8 a.m. to 2 p.m., either on day 25 (i.e., 10 days after stopping MPP^+^ infusion) or on day 45 (i.e., 30 days after stopping MPP^+^ infusion) (see Figure 1A). Rats were sacrificed within 30 min after behavioral testing, by decapitation after deep anesthesia with 2-bromo-2-chloro-1,1,1-trifluoroethane (halothane from Sigma; no reaction to handling or tail pinch, while still breathing).

To test the impact of the A_2A_ receptor antagonist SCH58261 (Tocris, Bristol, UK), it was administered intraperitoneally daily at 7 p.m., either from day 15 to day 24 or from day 35 to day 44 (see Figure 1). We selected a dose of SCH58261 of 0.1 mg/kg that was previously shown to afford neuroprotection in different animal models of brain diseases (e.g., [12,26,27,28,33]). Since we have previously shown that SCH58261 does not affect the motor behavior of rodents (see [33,98]), we did not test the impact of SCH58261 on control rats but only tested the ability of SCH58261 to attenuate the effects of MPP^+^. 

Behavioral tests were performed as previously described [12,33,97,99], in a sound-attenuated room maintained at 21–23 °C and 50–60% humidity with red lightening (8 lux light intensity), to which the animals were previously habituated for at least 1 h before beginning behavioral tests. The tests were video-recorded and analyzed with the ANY-maze Video Tracking Software (version 6.18; Stoelting, Wood Dale, IL, USA). The apparatuses were cleaned with 10% ethyl alcohol to remove odors after testing each rat. In the open-field test, rats were allowed to freely explore a wooden arena (100 × 100 cm, gray walls and gray floor) for 15 min to assess their spontaneous locomotor activity by quantifying the total distance traveled and the number of rearing events [12,33,97,99]. In the accelerated rotarod test, rats were tested for their balance and motor coordination in a single session, without previous pretraining, during which the cylinder rotation speed was progressively increased (from 16 to 37 rpm in 5 steps) to measure the latency to fall, as previously described [33,100]. We did not carry out behavioral tests specifically designed to score catalepsy or akinesia.

### 4.4. Quantification of Dopamine

DA was quantified by HPLC, essentially as previously described [12,100,101]. Striatal samples were homogenized by ultrasonication in 0.1 M perchloric acid with 0.02% sodium metabisulfite and 10 μM 3,4-dihydroxybenzylamine as internal standard. After centrifugation at 10,000× *g* for 5 min at 4 °C, the supernatant (20 μL) was separated through a LiChrospher 100 RP-18 (5 μm) cartridge (Merck, Lowe, NJ, USA) fitted into a Manu-cart holder (Merck) with a mobile phase (pH 4.0) consisting of 0.1 M KH_2_PO_4_, 3 mM octane-1-sulfonic acid sodium salt, 0.1 mM EDTA, and 10% (*v*/*v*) methanol, at a flow rate of 1.2 mL/min. The detection was achieved with a Coulochem-II electrochemical detector (Elsichrom AB, Knivsta, Sweden) with a dual-electrode analytical cell (ESA 5011A) and sensitivity at 0.5 nA/V, and the peak areas of the external standards were used to quantify DA levels.

### 4.5. Immunohistochemistry of Tyrosine Hydroxylase

The immunodetection of tyrosine hydroxylase (TH) was carried out in free-floating brain sections, as previously described [12,100,101]. Briefly, rats were anesthetized with sodium thiopental and transcardially perfused with ice-cold phosphate-buffered saline (PBS) followed by 4% paraformaldehyde in PBS. The brains were removed, post-fixed in 4% paraformaldehyde for 16–24 h, and cryoprotected in 30% sucrose for 48 h at 4 °C. The brain was then frozen in dry ice, and 50 μm coronal sections were prepared using a cryostat (Leica CM3050S, Wetzlar, Germany) at −21 °C. The sections were washed 3 times for 10 min with PBS and incubated with PBS supplemented with 10% methanol and 1.05% hydrogen peroxide for 40 min at room temperature (RT) to block endogenous peroxidase-like activities. After washing 3 times for 10 min with PBS and blocking endogenous proteins with 10% normal goat serum in PBS supplemented with Triton X-100 (blocking solution) for 2 h at RT, the sections were incubated with the primary antibody (rabbit anti-TH, 1:1000; from Merck-Millipore, Lisboa, Portugal) diluted in a blocking solution at 4 °C for 48 h. The sections were then washed with PBS before incubation for 2 h at RT with a secondary goat anti-rabbit biotinylated antibody (1:200, Vector Labs, Newark, CA, USA) diluted in blocking solution and washed with PBS. The avidin–biotin–horseradish peroxidase conjugate (ABC Staining System, Santa Cruz Biotechnology, Dallas, TX, USA) was used for 40 min at RT for amplification of the signal, which was revealed with a DAB Peroxidase Substrate Kit (Vector labs). The reaction was stopped by washing with PBS before mounting on gelatin-coated slides, which were dried, dehydrated by a gradient of ethanol, and cleared with xylene. Finally, the sections were cover-slipped with Entellan (Merck). The stained brain sections were visualized using a Zeiss Imager Z2 fluorescence microscope (Zeiss, Jena, Germany) equipped with an AxioCam HRm camera (Zeiss). Immunoreactivity was measured by semi-quantitative densitometric analysis using an image-analysis program (ImageJ software, version 1.53k). The optical densities of the control group were averaged, and the values of other groups were calculated as a percentage of that mean.

### 4.6. Western Blot Analysis

The analysis of the density of different synaptic markers was carried out by Western blotting [102,103] in total membrane extracts from the striatum prepared as previously described [104]. Briefly, one-half of the ipsilateral striatum was homogenized at 4 °C in sucrose solution (0.32 M) containing 50 mM Tris-HCl, 2 mM EGTA, and 1 mM dithiothreitol, pH 7.6. This homogenate was centrifuged at 3000× *g* for 10 min at 4 °C, and the supernatants were collected and centrifuged at 21,000× *g* for 20 min at 4 °C. The pellet was taken as the total membrane fraction and was resuspended in RIPA (50 mM Tris, 150 mM NaCl, 1% IGEPAL, 0.5% sodium deoxycholate, 1 mM EDTA, and 0.1% sodium dodecyl sulfate) plus cOmplete tablets EDTA-FREE Easy pack (Roche, Amadora, Portugal), 0.1 mM dithiothreitol, and 0.1 mM phenylmethylsulfonyl fluoride (PMSF). The amount of protein was determined using the Pierce bicinchoninic acid method (Thermo Fisher Scientific, Porto Salvo, Portugal), and samples were diluted to a final concentration of 2 μg protein/μL in SDS-PAGE buffer containing 30% (*v*/*v*) glycerol, 0.6 M dithiothreitol, 10% (*w*/*v*) sodium dodecyl sulfate, and 375 mM Tris-HCl, pH 6.8, and then boiled at 95 °C for 5 min. 

The samples and the prestained molecular weight markers (GE Healthcare, Carnaxide, Portugal) were separated by SDS-PAGE (10% with a 4% concentrating gel) under reducing conditions and electro-transferred to polyvinylidene difluoride membranes (0.45 μm, from GE Healthcare). After blocking for 2 h at RT with 5% milk in Tris-buffered saline, pH 7.6, containing 0.1% Tween 20 (TBS-T), the membranes were incubated overnight at 4 °C with the different antibodies, namely guinea-pig anti-vesicular GABA transporter (vGAT, 1:1000, from Calbiochem, San Diego, CA, USA), guinea-pig anti-vesicular glutamate transporter type 1 (vGluT1, 1:5000, from Chemicon, Merck-Millipore), and a rabbit anti-DAT (1:3000; from Chemicon). After four washing periods for 10 min with TBS-T containing 0.5% milk, the membranes were incubated with either alkaline phosphatase-conjugated anti-rabbit (1:20,000), anti-mouse secondary antibody (1:10,000), or horseradish peroxidase-conjugated secondary goat anti-rabbit antibody in TBS-T containing 1% milk for 90 min at RT. After five 10 min washes in TBS-T with 0.5% milk, the membranes were incubated with enhanced chemifluorescence for 5 min or with enhanced chemiluminescence (Amersham Biosciences, Little Chalfont, UK) before being analyzed with a VersaDoc 3000 (Bio-Rad, Amadora, Portugal) and quantified with the Quantity One software version 4.6.8 (Bio-Rad). The membranes were always re-probed to confirm the amount of loaded protein by measuring the immunoreactivity against β-actin using a mouse anti-β-actin antibody (1:20,000 dilution).

### 4.7. Viability of Synaptosomes

Striatal synaptosomes (purified synapses) were prepared as previously described [37,104]. After decapitation, half of ipsilateral striatum was dissected and homogenized in sucrose (0.32 M) solution containing 1 mM EDTA, 10 mM HEPES, and 1 mg/mL BSA (Sigma), pH 7.4 at 4 °C, supplemented with a protease inhibitor, PMSF (0.1 mM), a cocktail of inhibitors of proteases (CLAP 1%, Sigma), and dithiothreitol (1 μM). The homogenate was centrifuged at 3000× *g* for 10 min at 4 °C, and the resulting supernatant was further centrifuged at 14,000× *g* for 12 min at 4 °C. The resulting pellet was resuspended in 1 mL of a 45% (*v*/*v*) Percoll solution in HEPES buffer (140 mM NaCl, 5 mM KCl, 25 mM HEPES, 1 mM EDTA, and 10 mM glucose, pH 7.4). After centrifugation at 14,000× *g* for 2 min at 4 °C, the white top layer was collected (synaptosomal fraction), resuspended in 1 mL HEPES buffer, and further centrifuged at 14,000× *g* for 2 min at 4 °C. The pellet was then resuspended in Krebs-HEPES solution (in mM: NaCl 125, KCl 3, NaH_2_PO_4_ 1.25, MgCl_2_ 1, CaCl_2_ 2, HEPES 25, glucose 10, pH 7.4) for viability assays. The purity of this synaptic fraction has been previously quantified as >95% [105,106]. 

The activity of cellular dehydrogenases, taken as an index of cellular dysfunction [107], was estimated by the quantification of the reduction in the yellow MTT salt [3-(4,5-dimethylthiazole-2-yl)-2,5-diphenyltetrazolium bromide] to form an insoluble purple formazan dye, which can be measured spectrophotometrically at 570 nm. Striatal synaptosomes (0.5 µg/µL) were incubated for 2 h at 37 °C in Krebs-HEPES solution without or with the different concentrations of MPP^+^ (50, 100, 150, or 200 µM) and without or with SCH58261 (50 nM). MTT (10 μL of a 5 mg/mL solution in PBS) was then added, and the mixture was incubated for 1 h at 37 °C in Krebs-HEPES solution before centrifugation at 12,000× *g* for 2 min. The resulting pellet was resuspended in 300 μL of acidic (0.04 M HCl) isopropanol and incubated for 30 min in a rotating shaker. After centrifugation at 12,000× *g* for 2 min, 100 μL of the supernatant was then used for spectrophotometric analysis at 570 nm.

Cellular damage was evaluated by measuring the amount of cytoplasmic LDH released into the medium, using an assay based on the reduction in NAD^+^ by the action of LDH, as previously described [37,104]. Striatal synaptosomes (0.5 µg/µL) were incubated for 2 h at 37 °C in Krebs-HEPES solution without or with the different concentrations of MPP^+^ (50, 100, 150, or 200 µM) and without or with SCH58261 (50 nM). An additional aliquot of striatal synaptosomes was boiled at 95 °C for 5 min (total disruption of nerve terminals, functioning as positive control). After centrifugation at 12,000× *g* for 5 min at 4 °C, the supernatants were collected for analysis, and the pellets were resuspended in 200 µL of a hypotonic solution (15 mM Tris, pH 7.4). LDH activity was determined spectrophotometrically (for 120 s at 340 nm) at 30 °C in samples of the supernatant and of the pellet (80 µL) in 2.5 mL Tris/NaCl buffer (81.3 mM Tris and 203.3 mM NaCl, pH 7.20 at 30 °C) containing 0.20 mM NADH and 1.50 mM pyruvate. 

### 4.8. Statistical Analyses

The values are presented as mean ± S.E.M. with the number of determinations (n, preparations from different rats). A Grubbs test was first used to detect putative outliers. The comparison of two experimental conditions was performed using a two-tailed Student’s *t*-test with Welsh correction. Otherwise, statistical analysis was performed by one-way analysis of variance (ANOVA), followed by Tukey’s multiple comparison post hoc test; *p* < 0.05 was considered to represent statistical significance. Statistical analysis was performed using GraphPad Prism software (version 6.0; GraphPad Software, La Jolla, CA, USA).

## 5. Conclusions

It is concluded that the intracerebral MPP^+^ slow infusion triggers an early striatal synaptotoxicity before the onset of behavioral measurable motor dysfunction, which is accompanied by the overt neurodegeneration of DA neurons in the substantia nigra. The A_2A_R blockade affords dual neuroprotection, of both striatal synaptotoxicity during the prodromic phase of this PD model and the overt nigra neurotoxicity during the onset of PD. The behavioral neuroprotection afforded by the A_2A_R blockade is more robust when controlling synaptotoxicity rather than neurotoxicity, prompting future studies to investigate the properties of novel anti-PD A_2A_R antagonists with respect to synaptic and nonsynaptic A_2A_R to fully exploit their neuroprotective potential and highlighting the need to understand the different signaling mechanisms operated by A_2A_R in different cellular compartments. 

## Figures and Tables

**Table 1:** Neurochemical and behavioral parameters are presented as mean ± SEM. Statistical values in blue represent significance between vehicle and MPP^+^ (black are nonsignificant differences), whereas values in red represent significance between MPP^+^ and MPP^+^ + SCH groups (black are nonsignificant differences). SCH: SCH58261.

	Vehicle	MPP^+^	MPP^+^ + SCH	Statistics
**After 25 days (n = 8)**				
DA levels (pmol/mg tissue)	84.2 ± 3.48	53.7 ± 3.10		*p * < 0.0001
TH immunoreactivity (% vehicle)	100	95.5 ± 3.39		*p* = 0.033
total distance traveled (m)	21.9 ± 0.62	21.6 ± 0.73		*p* = 0.446
number of rearing events	9.63 ± 0.50	9.50 ± 0.78		*p* = 0.895
latency to fall (s)	172 ± 5.52	159 ± 5.98		*p* = 0.155
**After 45 days (n = 8)**				
DA levels (pmol/mg tissue)	86.4 ± 3.27	12.6 ± 2.45		*p * < 0.0001
TH immunoreactivity (% vehicle)	100	60.4 ± 4.65		*p * < 0.0001
total distance traveled (m)	21.2 ± 0.73	13.9 ± 0.53		*p * < 0.0001
number of rearing events	9.87 ± 0.61	5.87 ± 0.55		*p * = 0.0003
latency to fall (s)	172 ± 5.17	98.0 ± 6.86		*p * < 0.0001
**After 45 days (n = 8); SCH 15–24 days**				
DA levels (pmol/mg tissue)	84.4 ± 2.65	11.8 ± 1.93	54.3 ± 1.90	*p * < 0.0001
TH immunoreactivity (% vehicle)	100	61.2 ± 2.69	79.0 ± 3.10	*p * = 0.002
total distance traveled (m)	22.0 ± 0.70	13.0 ± 0.57	18.9 ± 0.54	*p * < 0.0001
number of rearing events	10.8 ± 0.53	5.63 ± 0.46	8.13 ± 0.44	*p * = 0.0045
latency to fall (s)	176 ± 4.54	95.3 ± 3.91	138 ± 6.67	*p * = 0.0005
**After 45 days (n = 8); SCH 35–44 days**				
DA levels (pmol/mg tissue)	86.2 ± 3.54	9.06 ± 1.67	25.5 ± 3.42	*p * = 0.004
TH immunoreactivity (% vehicle)	100	61.7 ± 2.43	72.6 ± 1.86	*p * = 0.009
total distance traveled (m)	21.9 ± 0.70	13.1 ± 0.41	17.0 ± 0.52	*p * = 0.0002
number of rearing events	10.0 ± 0.63	5.38 ± 0.38	6.25 ± 0.45	*p* = 0.390
latency to fall (s)	173 ± 5.79	100 ± 5.12	132 ± 3.91	*p * = 0.007

## Data Availability

The data that support the findings of this study are available from the corresponding author upon reasonable request.

## Abbreviations

A_2A_R, adenosine A_2A_ receptors; DA, dopamine; DAT, dopamine transporter; icv, intracerebroventricular; i.p., intraperitoneal; LDH, lactate dehydrogenase; MPP^+^, 1-methyl-4-phenylpyridinium; MPTP, 1-methyl-4-phenyl-1,2,3,6-tetrahydropyridine; MTT, 2,5-diphenyl-2H-tetrazolium; PBS, phosphate-buffered saline; PD, Parkinson’s disease; PMSF, phenylmethylsulfonyl fluoride; RT, room temperature; SCH58261, 2-(2-furanyl)-7-(2-phenylethyl)-7H-pyrazolo[4,3-e][1,2,4]triazolo[1,5-c]pyrimidin-5-amine; TH, tyrosine hydroxylase; vGAT, vesicular GABA transporter; vGluT1, vesicular glutamate transporter type 1.
